# Novel Sulfated Oligosaccharide DP9 from Marine Algae, *Gracilaria lemaneiformis*: A Potent Galectin-3 Inhibitor for Pancreatic Cancer Therapy

**DOI:** 10.3390/md23110423

**Published:** 2025-10-30

**Authors:** Pingting Liu, Fengyuan Li, Zhicong Liu, Yang Liu

**Affiliations:** Guangdong Provincial Key Laboratory of Marine Biotechnology, Guangdong Engineering Technology Research Center of Offshore Environmental Pollution Control, Department of Biology, College of Science, Shantou University, Shantou 515063, China; 23ptliu@stu.edu.cn (P.L.); 23fyli@stu.edu.cn (F.L.); 17zcliu2@stu.edu.cn (Z.L.)

**Keywords:** oligosaccharide DP9, Gal-3 inhibitor, pancreatic cancer, BxPC-3 cells, Gal-3/EGFR/AKT/FOXO3 pathway

## Abstract

Galectin-3 (Gal-3) is a histologic marker of pancreatic cancer and a potential therapeutic target. This study aimed to characterize a novel sulfated agarose-derived oligosaccharide (DP9) from marine algae, *Gracilaria lemaneiformis*, evaluate its Gal-3 inhibitory activity, and investigate its anti-pancreatic cancer mechanisms. Through controlled acid hydrolysis, a series of odd-numbered oligosaccharides (DP3-11) were obtained, in which DP9 showed the strongest Gal-3 inhibition in hemagglutination assays. Structural analysis confirmed DP9’s unique composition including an alternating β (1→4)-D-galactose and α (1→3)-3,6-anhydro-L-galactose backbone, featuring partial 6-O-methylation on β-D-galactose and 6-O-sulfation on 3,6-anhydro-α-L-galactose residues. Molecular docking revealed DP9’s binding to Gal-3’s carbohydrate recognition domain through key hydrogen bonds (His158, Arg162, Lys176, Asn179 and Arg186) and hydrophobic interactions (Pro117, Asn119, Trp181 and Gly235), with the sulfate group enhancing binding affinity. In vitro studies demonstrated DP9’s selective anti-pancreatic cancer activity against BxPC-3 cells, including inhibition of cell proliferation; S-phase cell cycle arrest; induction of apoptosis; and suppression of migration and invasion. Mechanistically, DP9 attenuated the Gal-3/EGFR/AKT/FOXO3 signaling pathway while showing minimal cytotoxicity to normal cells. This study first demonstrated that agarose-derived odd-numbered oligosaccharides (DP9) can serve as effective Gal-3 inhibitors, which proved its potential as a marine oligosaccharide-based therapeutic agent for pancreatic cancer.

## 1. Introduction

Galectin-3 (Gal-3) is a 30 kDa β-galactoside-binding protein comprising three structural domains: an N-terminal domain, a tandem repeat domain, and a C-terminal carbohydrate recognition domain. Elevated Gal-3 expression is strongly associated with cancer progression and metastasis [1], especially highly expressed in stage I pancreatic cancer without lymph node metastasis [2]. Galectin-targeted inhibitors are developed given Gal-3’s specific affinity for β-galactosides.

Current Gal-3 inhibitors include small-molecule carbohydrates, natural polysaccharides, and their synthetic derivatives [3]. These compounds primarily exert antitumor effects by disrupting Gal-3-mediated cellular processes including adhesion, proliferation, and migration. Notably, the carbohydrate-based inhibitor β-pectin has demonstrated clinical efficacy, particularly given that combined therapy involving β-pectin with the anti-PD-1 (Programmed Cell Death Protein 1) antibody pembrolizumab demonstrated clinical activity in metastatic melanoma and head/neck squamous cell carcinoma [4,5]. Another promising candidate, GB1211, has shown positive outcomes in Phase II trials for advanced liver cancer [6]. Natural glycans with rich β-galactosidic bonds had a general affinity to Gal-3 and inhibited its biological functions. A recent study showed that Gal-3 inhibitors effectively inhibit pancreatic ductal adenocarcinoma tumor growth and metastasis in vivo [7]. Polysaccharides isolated from Panax notoginseng were bound to Gal-3. thus inactivating downstream pathways [8]. HH1 polysaccharide extracted from safflower blocked the Gal-3/EGFR/AKT/FOXO3 signaling pathway [9].

The polysaccharides from marine algae, *Gracilaria lemaneiformis* (*G. lemaneiformis*), are rich in agar, with plenty of β-galactosidic bonds, which have been studied by our laboratory for years [10,11,12,13,14]. Moreover, the agaro-oligosaccharide bioactivities, including prebiotic, antioxidation, anti-inflammatory, hepatoprotective functions, have been the hotspots over the past two decades [15,16,17,18]. Here, the presence of oligosaccharides from *G. lemaneiformis* would be determined as the research object to search for a novel nature Gal-3 inhibitor. Polysaccharides from *G. lemaneiformis* are mainly composed of repeated units of β-D-galactose and 3,6-anhydrous α-L-galactose with rich β-galactosidic bonds and few sulfate residues. Thus, agaro-oligosaccharides have two oligosaccharide types, which have α-1,3-linked-3,6-anhydro-L-galactose and β-1,4-linked-D-galactose in nonreducing ends. Several existing methods for hydrolyzing agarose to agaro-oligosaccharides include acidolysis, enzymolysis and fermentation methods [19,20,21]. Acidolysis using hydrochloric acid or sulfuric acid is commonly implemented to obtain agaro-oligosaccharides because acidolysis is cheap, non-specific and easy to operate without any boiling or a water bath to dissolve agarose during acidolysis. Moreover, a relatively high concentration of agarose could be degraded into agaro-oligosaccharides with polysaccharide fragments containing novel structures. To develop a novel nature Gal-3 inhibitor, it is necessary to further research the structure–function relationship of agaro-oligosaccharides in detail.

Oligosaccharides derived from *G. lemaneiformis* were selected as the research object; they were purified and identified based on their affinity with Gal-3. Based on the molecular docking methods used, the oligosaccharides and Gal-3 binding were analyzed in detail. Finally, the various anti-pancreatic cancer activities of the oligosaccharides as the Gal-3 inhibitor would be detected and confirmed in vitro. Special focus will be placed on their ability to modulate the specific signaling pathway, establishing structure–activity relationships for these potential therapeutic agents.

## 2. Results

### 2.1. Separation and Identification of Acidolysis Oligosaccharides from G. lemaneiformis

**Figure 1:**
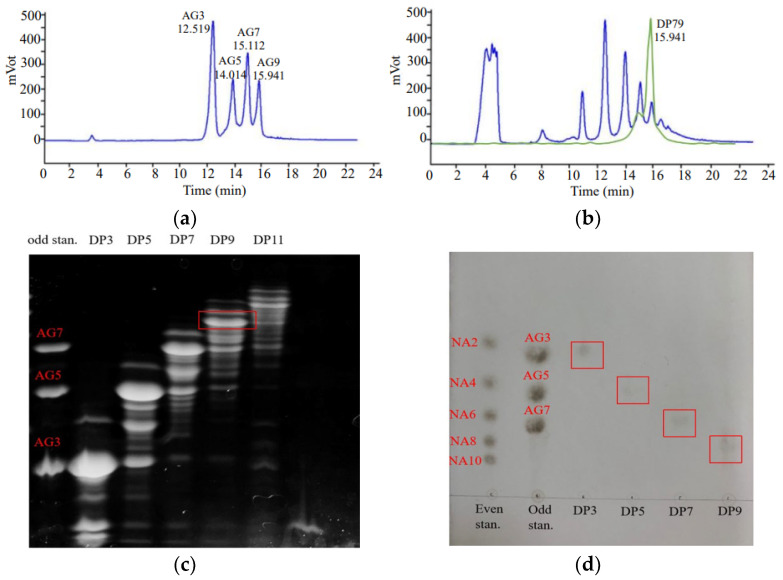
Separation and purification of oligosaccharides. (**a**) The mixed standards of AG3, AG5, AG7 and AG9 in HPLC-ELSD detection. (**b**) The results of HPLC-ELSD detection of mixed oligosaccharides after acid hydrolysis and separation of DP9 (green line) from *G. lemaneiformis*. (**c**) The result of FACE of the isolated oligosaccharides from *G. lemaneiformis*. (**d**) The result of thin-layer chromatography (TLC) of the isolated oligosaccharides from *G. lemaneiformis*.

Figure 1a illustrates the effective separation of agaro-oligosaccharide standards (AG3, AG5, AG7, and AG9), where oligosaccharides with lower molecular weights (e.g., AG3) eluted earlier than their higher-molecular-weight counterparts (AG5, AG7, and AG9). The first peak at 12.519 min corresponded to AG3, the smallest oligosaccharide, while subsequent peaks represented progressively larger oligosaccharides (AG5, AG7, and AG9). Figure 1b presents a chromatogram of mixed oligosaccharides obtained via *G. lemaneiformis* acid hydrolysis, along with the DP9 fraction isolated through chromatography. The acidolysis products (DP3-DP11) and purified DP9 exhibited retention times similar to those of the agaro-oligosaccharide standards (AG3-AG9). This retention time alignment confirmed that the DP9 fraction from *G. lemaneiformis* consisted of oligosaccharides with approximately nine degrees of polymerization.

To further characterize the oligosaccharide components derived from *G. lemaneiformis* and identify potential bioactive candidates, we analyzed FACE, TLC and Gal-3-mediated hemagglutination assays of the acidolysis products. Figure 1c clearly demonstrates the effective separation of ANTS-labeled oligosaccharides from *G. lemaneiformis* through FACE. Electrophoretic analysis showed that DP3 exhibited identical mobility to the standard AG3 and the DP5/AG5 pair, while the DP7/AG7 pair also displayed the same migration characteristics. The oligosaccharides (DP3, DP5, DP7, DP9, DP11) showed excellent resolution from each other, with the target products displaying the most intense fluorescent bands. Moreover, the TLC results of various oligosaccharides in Figure 1d showed similar results. DP9, as an odd-numbered oligosaccharide, migrated between the even-numbered standards NA8 and NA10, while maintaining clear separation from both adjacent markers and other DP species. This differential migration pattern between odd- and even-numbered oligosaccharides confirmed the method’s resolution capacity for carbohydrates of varying polymerization degrees.

The biological relevance of these findings was further supported by Gal-3-mediated hemagglutination assays (Figure S1). Among the odd-numbered oligosaccharides (DP3-DP11) obtained from acid hydrolysis, DP9—the nonasaccharide composed of nine repeating units—exhibited the strongest inhibitory activity (lowest inhibitory concentration) among both the odd-numbered oligosaccharides (DP3-DP11) and the even-numbered oligosaccharides (DP2-DP10 obtained through enzymatic hydrolysis; their TLC results are displayed in Figure S1. This prominent bioactivity, combined with its high purity, as demonstrated by the separation profile, led to its selection as the primary candidate for subsequent investigations.

### 2.2. Analysis of DP9 Structural Characteristics

**Figure 2:**
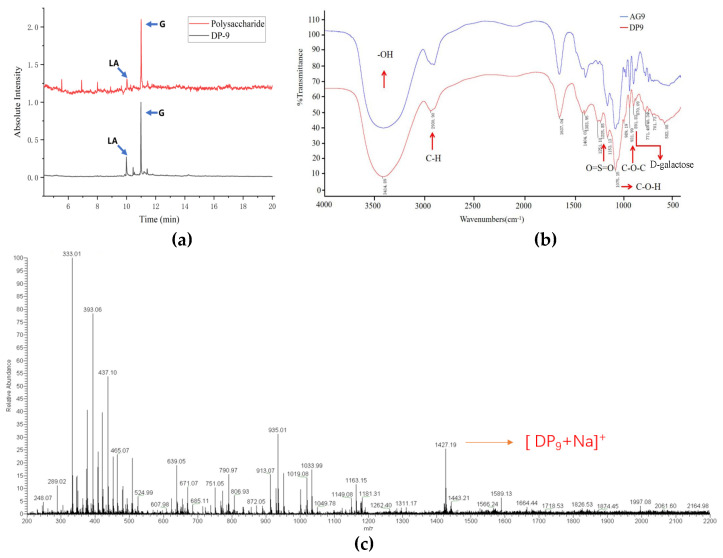
Structure characterization of DP9. (**a**) GC-MS results of AnGal/Gal ratios in polysaccharide and DP9, LA: 3,6-anhydro-L-galactose. (**b**) The FTIR (Fourier-transform infrared spectroscopy) results for DP9 and AG9. (**c**) The ESI-MS result for DP9.

The structural composition of DP9 was systematically characterized through GC-MS analysis, FTIR spectroscopy, and mass spectrometry. First, analysis of the monosaccharide composition of DP9 (Supplementary Material S3) confirmed galactose as the predominant monosaccharide constituent. Figure 2a shows polysaccharide AnGal:Gal at 1:3.4, in agreement with Xie et al.’s findings (1:3.1) [13]. In DP9, the ratio shifted to 1:3.0, suggesting acid that hydrolysis increased the presentation of AnGal (needs NMR). High-temperature acidic preparation likely degraded AnGal’s strained ether ring in both, causing content underestimation and lower GC-MS values compared to the theoretical 1:1 ratio.

FTIR spectroscopic analysis (Figure 2b) provided further structural insights for DP9, displaying characteristic absorption bands consistent with agaro-oligosaccharides. The broad peak at 3406.77 cm^−1^ corresponded to hydroxyl stretching vibrations, indicative of extensive hydrogen bonding. Diagnostic peaks at 1075.04 cm^−1^ (glycosidic bonds) and 931.76 cm^−1^ (3,6-anhydro-L-galactose C-O-C) were observed, while the absence of bands at 1243 cm^−1^ and 847 cm^−1^ confirmed the lack of sulfate groups. These spectral features matched previous reports [22] and supported the proposed disaccharide repeating unit structure.

Mass spectrometric analysis conclusively determined the molecular composition. Based on the established structural motif (G-A-G-A-G-A-G-A-G) and previous characterization of DP3 [23], the theoretical *m*/*z* for [DP9 + Na]^+^ was calculated as 1427.32. The ESI-MS spectrum (Figure 2c) showed excellent agreement with this prediction, displaying a predominant molecular ion peak at *m*/*z* 1427.19. This result confirmed DP9’s composition as five galactose and four 3,6-anhydro-L-galactose residues, validating both the proposed structure and high purity of the isolated oligosaccharide.

### 2.3. Structural Elucidation of DP9 by Multidimensional NMR Spectroscopy

**Figure 3:**
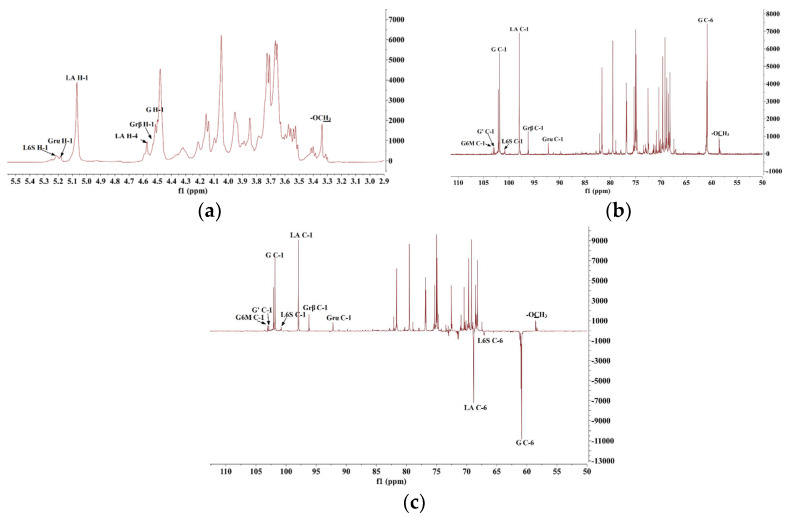
One-dimensional NMR spectrum for DP9. (**a**) ^1^H-NMR spectrum (suppression of water signal). (**b**) ^13^C-NMR spectrum. (**c**) DEPT-135 spectrum. For a summary of all other chemical shifts, refer to Table 1.

**Figure 4:**
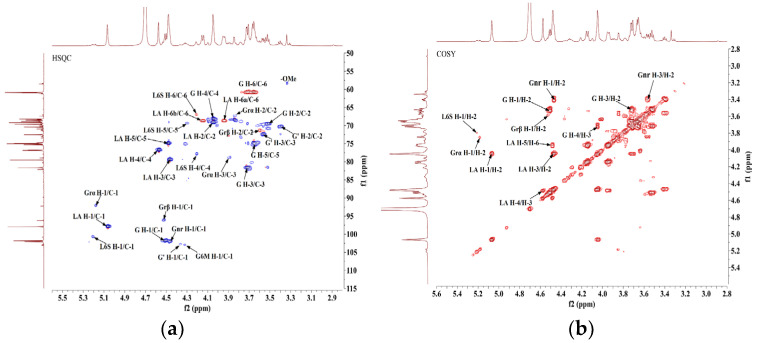

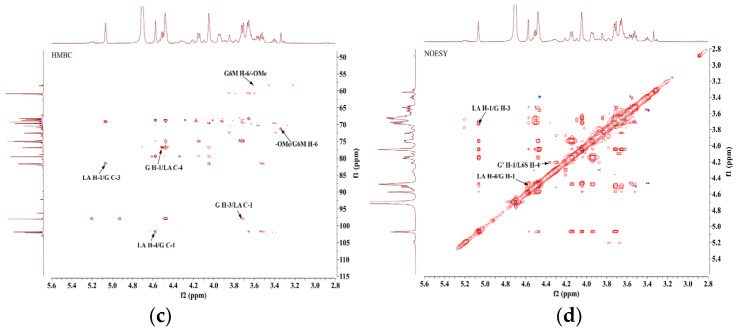
Two-dimensional NMR spectra for DP9. (**a**) HSQC spectrum. (**b**) COSY spectrum. (**c**) HMBC spectrum. (**d**) NOESY spectrum. For summary of all other chemical shifts, refer to Table 1.

The structural characterization of DP9 was comprehensively investigated through 1D (1H-NMR, 13C-NMR and DEPT-135 in Figure 3) and 2D (COSY, HSQC, HMBC and NOESY in Figure 4) NMR techniques. The 1H-NMR spectrum (Figure 3a) displayed overlapping proton resonances in the δ 3.0–5.5 ppm region, and multiple anomeric proton resonances in the range of δ 4.2–5.5 ppm [24], indicating the presence of multiple sugar residues with partial overlapping. In contrast, the 13C-NMR spectrum (Figure 3b) revealed characteristic anomeric carbon signals (δ 90–110 ppm), confirming the presence of multiple sugar residues. These observations, consistent with previous reports [25,26], supported DP9’s identity as a red algal polysaccharide containing galactose and 3,6-anhydrogalactose (AnGal) units. The distinctive AnGal C-1 chemical shift at δ 98.06 (within 95–100) ppm (Figure 3b) further verified the L-configuration typical of red algal agarose polysaccharide [27,28]. DEPT-135 (Figure 3c) analysis facilitated the identification of carbon types, including primary, secondary, tertiary and quaternary carbon. Figure 3c displays prominent negative signals at δ 59–63 ppm, indicating the presence of -CH2 groups. Combined with monosaccharide composition analysis, these signals are assigned to the C-6 positions of sugar residues. Notably, distinct inverted peaks (positive signals) are observed in the δ 65–70 ppm region, suggesting substitution at the C-6 positions of the sugar residues in DP9 [29].

By comprehensively analyzing 2D NMR spectra (HSQC, COSY, HMBC, and NOESY) of DP9 (Figure 4) and integrating monosaccharide composition data from the literature [29,30,31,32,33,34,35], the glycosidic linkages and chemical shifts of DP9 were systematically assigned as follows:

(1) Reductive Galactose Terminal Residues (Grα/Grβ) [31,34]: Cross-peaks at δ 5.18/92.37 ppm (H-1/C-1) and δ 4.52/96.15 ppm (H-1/C-1) in the HSQC (Figure 4a) anomeric region, consistent with α/β-configured reducing galactose terminals (Correc et al., 2011), were assigned to →3)-α-D-Galp (Grα) and →3)-β-D-Galp (Grβ), respectively. Chemical shifts were confirmed via COSY (Figure 4b)/HSQC.
(2) →4)-α-L-AnGalp-(1→ Residue (LA) [29,34]: A strong HSQC cross-peak at δ 5.06/98.06 ppm (H-1/C-1) matched α-L-AnGalp [28,29,30]. Sequential assignment via COSY/HSQC revealed H-1–H-5 shifts (δ 5.06, 4.05, 4.46, 4.57, 4.48 ppm) and C-1–C-6 shifts (δ 98.06, 69.11, 79.63, 76.89, 74.91, 68.72 ppm). Downfield shifts at C-1, C-3, C-4, and C-6 indicated →4)-α-L-AnGalp-(1→ (LA).
(3) β-D-Galp Residues (G/Gnr) [30]: Cross-peaks at δ 4.51/101.84 ppm and δ 4.46/102.12 ppm (H-1/C-1) corresponded to β-Galp [25,31,32,33,34,35]. For δ 4.51/101.84 ppm, H-1–H-5 shifts (δ 4.51, 3.53, 3.72, 4.04, 3.65 ppm) and C-1–C-6 shifts (δ 101.84, 69.74, 81.70, 68.18, 75.03, 60.92 ppm) suggested →3)-β-D-Galp-(1→ (G). The δ 4.46/102.12 ppm signal was assigned to →4)-α-L-Galp6S-(1→ (L6S) with sulfation at C-6. The G→L6S linkage was weaker than G→LA, with the H-1 integral ratio (L6S:LA = 1:8.20), indicating G→LA as the dominant repeating unit.
(4) Sulfated and Methylated Modifications [32,33,34]: The absence of cross-peaks in HSQC non-anomeric regions (δ 4.65–5.10 ppm/δ 60–90 ppm) ruled out sulfation at β-D-Galp C-2 or α-L-AnGalp C-2. A weak cross-peak at δ 5.21/100.80 ppm (H-1/C-1) was assigned to →4)-α-L-Galp6S-(1→ (L6S). A cross-peak at δ 3.33/58.57 ppm (-OMe) indicated β-D-Galp C-6 methylation [25,31,34,35] assigned to →4)-α-L-Galp6S-(1→ (G6M).

The above key assignments of ^1^H and ^13^C chemical shifts for each sugar residue in DP9 are summarized in Table 1. Moreover, the glycosidic linkages of DP9 were deduced [29,34,35] from the HMBC spectrum (Figure 4c) and NOESY spectrum (Figure 4d) as follows:

(1) HMBC correlations: G-H1 (δ 4.51) ↔ LA-C4 (δ 76.89), LA-H4 (δ 4.57) ↔ G-C1 (δ 101.84); NOESY correlation: LA-H4 ↔ G-H1, indicating →3)-β-D-Galp-(1→4)-α-L-AnGalp-(1→ (G→LA).
(2) HMBC correlations: LA-H1 (δ 5.06) ↔ G-C3 (δ 81.70), G-H3 (δ 3.72) ↔ LA-C1 (δ 98.06); NOESY correlation: LA-H1 ↔ G-H3, indicating →4)-α-L-AnGalp-(1→3)-β-D-Galp-(1→ (LA→G).
(3) NOESY correlation: G’-H1 (δ 4.37) ↔ L6S-H4 (δ 4.20), indicating →3)-β-D-Galp-(1→4)-α-L-Galp6S-(1→ (G→L6S).

**Figure 5:**
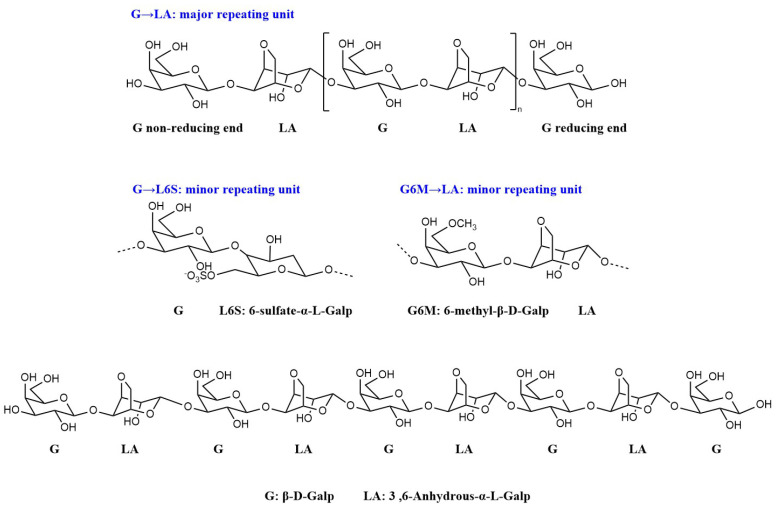
Repeating units and supposed structure of DP9 based on the detection results.

Based on the above analysis, the DP9 sample is primarily composed of a disaccharide repeating unit consisting of a β-D-galactose residue (G) linked at the 1,3-position and an α-L-3,6-anhydrogalactose residue (LA) linked at the 1,4-position. A minor component is a disaccharide unit comprising a β-D-galactose residue linked at the 1,3-position and an α-L-galactose-6-sulfate residue linked at the 1,4-position (L6S). Additionally, a small proportion of the β-D-galactose residues exhibit O-6 methylation substitution (G6M). The proposed repeating structural model is shown in Figure 5.

Here, as the terminal monosaccharide of an oligosaccharide chain, if it is the reducing end, its anomeric carbon (C1) must possess a free hemiacetal group (-OH). This group can interconvert between the open-chain aldehyde form and the cyclic hemiacetal form in solution, conferring reducibility. When LA is forced to occupy the reducing end, its C1 must expose the unstable hemiacetal structure. However, the inherent strain of the internal ether ring (a five-membered oxygen-containing ring) in the LA molecule renders it highly sensitive to this state. Under acidic conditions or upon heating, the exposed hemiacetal group triggers rapid degradation of the LA molecule itself. The most common degradation pathway involves β-elimination [15,36]. Protonation of the hemiacetal oxygen by acid leads to cleavage of the C4-O bond, accompanied by the departure of the C5-H proton, resulting in the formation of a double bond between C4 and C5. Consequently, a 5-dehydro-L-furanone molecule is generated and cleaved from the oligosaccharide chain, thereby disrupting the LA unit.

### 2.4. Molecular Docking and Dynamic Simulation

**Figure 6:**
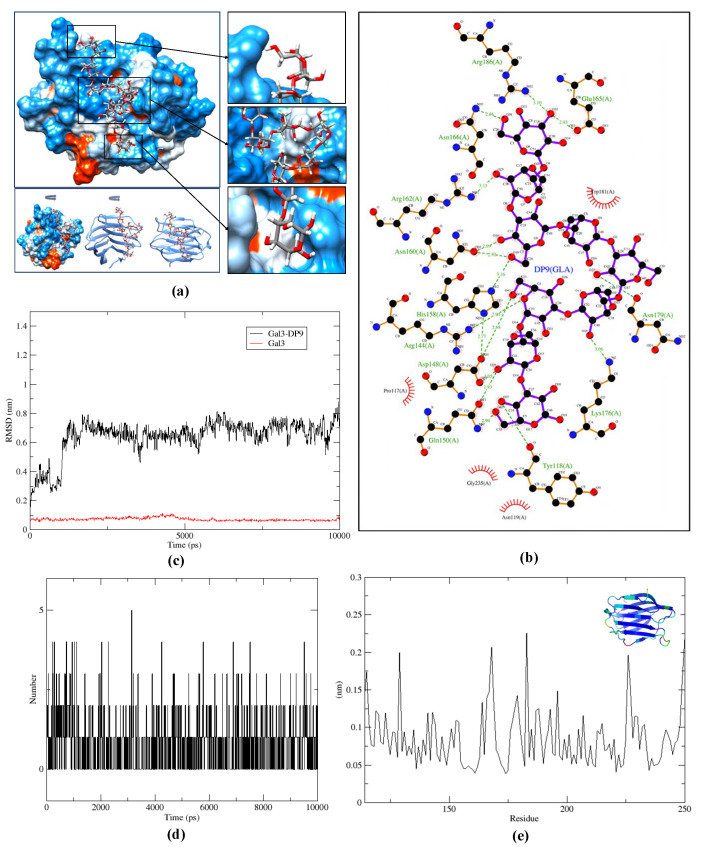
Binding conformation of docked DP9 and Gal-3 molecules. (**a**) Overview of DP9 and Gal-3 docking results (the upper left figure), different rotations for docking results (the lower left figure), DP9 non-reducing end presentation (the upper right figure), folded DP9 fragment (the middle right figure), and DP9 reducing end presentation (the lower right figure). (**b**) The 2D interaction included hydrogen bonds and hydrophobic interaction with key protein residues. (**c**) RMSD plot. (**d**) Hydrogen bond number against simulation period. (**e**) RMSF plot against residue sequence.

To validate the binding affinity of the major DP9 structure to Gal-3, molecular docking simulations were performed to explore the interactions between the DP9 oligosaccharide and Gal-3. The calculated Vina binding energy for DP9 was −6.7 kcal/mol, which was stronger than that of LacNAc (−5.3 kcal/mol) [37], as shown in Supplementary Table S3. The docking poses, illustrated in Figure 6a, demonstrated that DP9 was bound to the sugar-binding S-face of human Gal-3, consistent with previous reports [38], accompanied by noticeable folding of the protein surface upon ligand binding. Residue-level analysis revealed that several key interactions stabilized the DP9–Gal-3 complex, including hydrogen bonds formed between DP9 and Gal-3 residues His158, Arg162, Lys176, Asn179, and Arg186 (Figure 6b), as well as hydrophobic contributions from Pro117, Asn119, Trp181, and Gly235. These findings further confirm the critical role of Arg162 and Trp181 in mediating oligosaccharide–Gal-3 interactions, as previously suggested [37].

To assess the dynamic stability of the binding interaction, a 10 ns molecular dy-namics simulation was performed, and the resulting trajectory captured the DP9 recognition event by Gal-3. The root-mean-square deviation (RMSD) plot in Figure 6c showed that DP3 maintained stable binding to Gal-3 with only minor fluctuations (<0.3 Å deviation) after an initial equilibration phase, indicating a well-maintained ligand–protein interaction. The hydrogen bond dynamics illustrated in Figure 6d revealed significant fluctuations between 80 and 200 ps, which correlated with the observed RMSD increase during this period. Furthermore, the RMSF analysis in Figure 6e demonstrated that while Gal-3 exhibited higher flexibility in its loop and terminal regions, the backbone remained relatively stable, suggesting that the key residues involved in DP9 binding maintained their interactions throughout the simulation.

DP9 from *G. lemaneiformis* demonstrated significantly higher binding affinity to galectin-3 (Gal-3) compared to the natural inhibitor LacNAc. This was further validated by a 10 ns molecular dynamics simulation, which confirmed the stability of their interactions and underscored the importance of DP9 for potential inhibition efficacy studies. Notably, sulfite groups—present as minor repeating units in DP9—played a more substantial role in enhancing binding affinity to Gal-3 than methoxy groups, as evidenced by the comprehensive data presented in Table 2 and Table 3,  Tables S2 and S3 (Supplementary Materials).

**Table 2:** Chemical structures of DP9(G-LA) and DP9(G-L6S)1-DP9(G-L6S)6.

Name	Chemical Structure
DP9(G-LA)	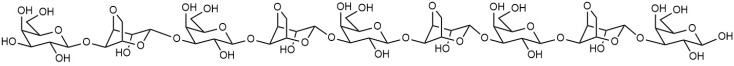
DP9(G-L6S)1	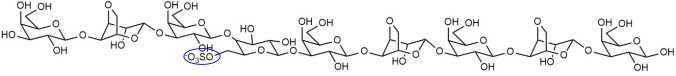
DP9(G-L6S)2	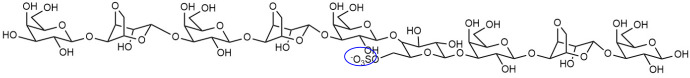
DP9(G-L6S)3	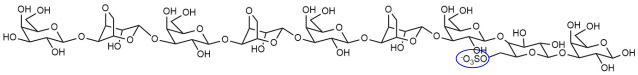
DP9(G-L6S)4	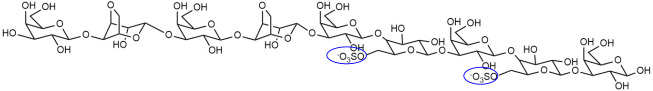
DP9(G-L6S)5	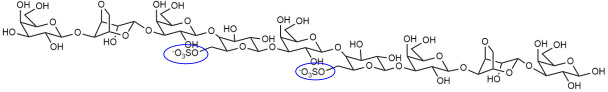
DP9(G-L6S)6	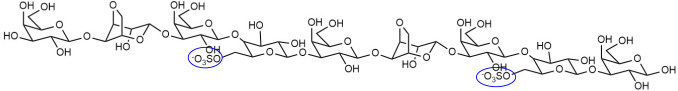

**Table 3:** Triplicate LeDock results docked to 1A3K.

Ligand Name	LeDock Score (kcal/mol)			
	Trial 1	Trial 2	Trial 3	Average
DP9(G-L6S)1	−9.07	−8.3	−8.14	−8.50
DP9(G-L6S)2	−7.55	−8.2	−7.38	−7.71
DP9(G-L6S)3	−9.35	−8.83	−7.83	−8.67
DP9(G-L6S)4	−8.84	−9.22	−9.44	−9.17
DP9(G-L6S)5	−9.43	−9.19	−7.72	−8.78
DP9(G-L6S)6	−9.77	−10.45	−10.05	−10.09
DP9(G-LA)	−8.82	−8.15	−8.4	−8.46

Detailed analysis in Table 3 revealed that the positions of sulfite substitutions on DP9 markedly influenced its binding affinity with Gal-3. Six DP9 (G-L6S) variants (with 1–6 sulfite modifications) were computationally predicted and docked, among which DP9(G-L6S)6 exhibited the lowest LeDock binding score, outperforming both DP9 (G-L6S)1–5 and the unmodified DP9 (G-LA). Strikingly, molecules with two sulfite substitutions consistently showed lower binding scores than those with a single substitution, indicating that the number of C6 sulfite modifications is a critical determinant of DP9–Gal-3 interactions. In their research, Mahanti et al. found that sulfur oxidation ligands strongly interact with Gal-3 both in structure and thermodynamics angels, by affinity-enhancing hydrogen bonds, and hydrophobic and π-interactions [39]. Among the single-substituted variants, C6 sulfite modification at the sixth monosaccharide position (DP9(G-L6S)2) yielded a higher binding score than modifications at the fourth (DP9(G-L6S)1) or eighth (DP9(G-L6S)3) positions, as well as the original DP9(G-LA) structure. Conversely, double substitutions at the fourth and eighth positions (DP9(G-L6S)6) achieved the lowest binding energy among all two-substituent variants. These findings demonstrate that edge-positioned C6 sulfite modifications (fourth and eighth monosaccharides) contribute more significantly to DP9-Gal-3 binding affinity than intermediate substitutions (e.g., the sixth position).

### 2.5. Anti-Pancreatic Cancer Activity of DP9

#### 2.5.1. Inhibitory Effect of DP9 on Angiogenesis and the Proliferation of Pancreatic Cancer BxPC-3 Cells In Vitro

**Figure 7:**
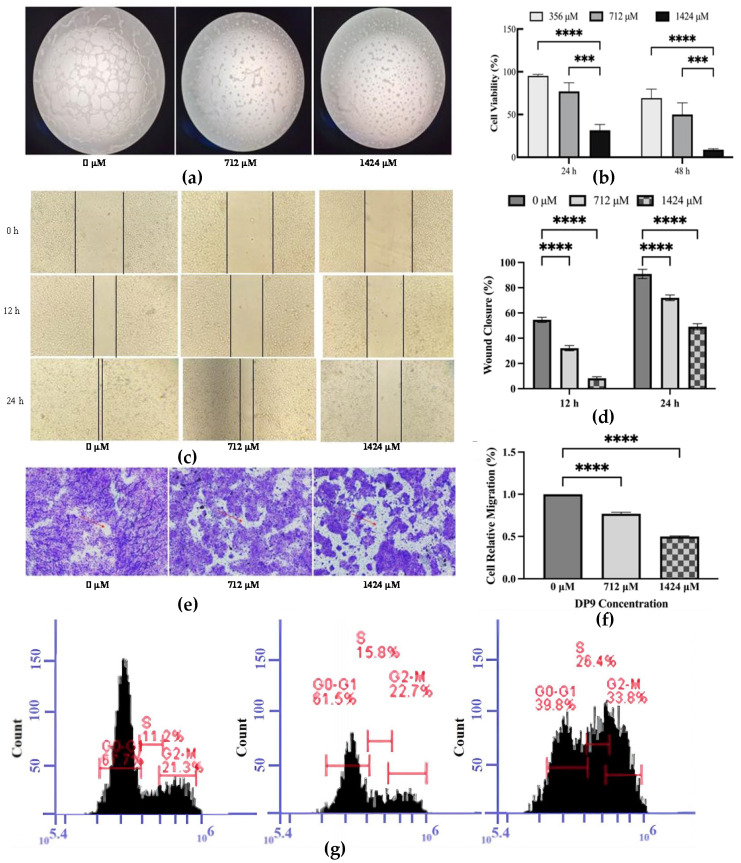
(**a**) Tube formation of BxPC-3 cells on Matrigel. (**b**) Cytotoxicity and proliferation effects of DP9 for BxPC-3 cells. (**c**,**d**): Effect of DP9 on scratch wound of BxPC-3 cells. (**e**,**f**): Effect of DP9 on BxPC-3 cell migration. (**g**) The cell cycle progression of BxPC-3 cells at 24 h incubated with different concentration of DP9. Data presented as average ± standard deviation (*n* = 3). (*n* = 3, *** *p* < 0.001 and **** *p* < 0.0001 compared with 356 µM in figure b, and compared with 0 µM in figure d–f, respectively).

To evaluate the antiangiogenic potential of DP9, a tube formation assay was performed. As shown in Figure 7a, DP9 exhibited potent antiangiogenic activity, reducing micro-vessel density in a dose-dependent manner. Specifically, treatment with 712 μM and 1424 μM DP9 significantly impaired the ability of BxPC-3 cells to form capillary-like structures compared to the control group (0 μM DP9), confirming its antiangiogenic effect in vitro.

As shown in Figure 7b, DP9 treatment significantly inhibited the proliferation of BxPC-3 pancreatic cancer cells in a concentration- and time-dependent manner. At 24 h, cell viability decreased from 95.17% (356 μM) to 77.04% (712 μM) and further to 31.58% (1424 μM). After 48 h of incubation, the viability dropped markedly to 69.22% (356 μM), 50.1% (712 μM), and only 8.82% (1424 μM). These results demonstrate that DP9 effectively suppresses BxPC-3 cell growth, with higher concentrations and longer exposure times leading to more pronounced inhibitory effects.

For comparison, the cytotoxicity and proliferation effects of DP9 on normal mouse fibroblast L929 cells are presented in Supplementary Figure S3.

#### 2.5.2. Inhibition Effect of DP9 on BxPC-3 Cell Migration

Given its anti-angiogenic activity, we further investigated whether DP9 could inhibit cell migration and invasion in the tumor microenvironment. The effect of DP9 on BxPC-3 cell migration was examined using a cell scratch assay (Figure 7c,d). At 0 h, identical central scratch areas were created across groups treated with 0 μM, 712 μM, and 1424 μM DP9. By 12 h, the scratch area expanded with increasing DP9 concentrations, resulting in decreased wound closure: the 0 μM group showed the smallest scratch area (fastest closure), while the 1424 μM group exhibited the largest remaining area. At 24 h, the scratch area in all groups was markedly reduced compared to at 0 h. Visual observation revealed near-complete closure in the 0 μM group, partial closure in the 712 μM group, and persistent large gaps in the 1424 μM group. Quantitative analysis (with the 0 h scratch area set as 100%) showed that at 12 h, the repaired area was 54.6%, 32.3%, and 8.3% for 0 μM, 712 μM, and 1424 μM DP9, respectively. By 24 h, these values increased to 91.7%, 71.4%, and 49%, respectively. These results demonstrate that DP9 significantly inhibits tumor cell migration. Supplementary Figure S4 further confirms that DP9 also suppresses cell invasion. Within the tested concentration range, higher DP9 concentrations correlated with stronger inhibition of tumor cell migration and invasion.

Since tumor cell invasion and metastasis require neovascularization to support new blood vessel formation, we evaluated DP9’s effect on cell migration using Transwell assays (Figure 7e,f). After 24 h of DP9 treatment, cell migration decreased to 87.02% and 51.73% at 712 μM and 1424 μM, respectively. The number of migrated cells showed a significant dose-dependent reduction with increasing DP9 concentrations.

#### 2.5.3. Effect of DP9 on S-Phase Arrest of BxPC-3 Cells

The effect of DP9 on cell cycle progression was evaluated in BxPC-3 cells following 24-h treatment. The results demonstrated that DP9 treatment significantly increased the proportion of cells in the S phase, accompanied by a concentration-dependent reduction in the percentage of cells in the G0-G1 and G2-M phases. These findings were further confirmed through histogram analysis of the cell cycle distribution (Figure 7g), which clearly showed that DP9 primarily inhibits BxPC-3 cell proliferation by inducing S-phase cell cycle arrest. Additional data on DP9’s effect on BxPC-3 cell apoptosis are presented in Supplementary Figure S5.

#### 2.5.4. Anti-Pancreatic Cancer Activity of DP9 Through Targeting the Gal-3/EGFR/AKT/FOXO3 Signaling Pathway

As a promising anti-pancreatic cancer drug candidate, DP9 demonstrates binding affinity to Gal-3 and inhibitory effects on the Gal-3/EGFR signaling pathway. To elucidate the molecular mechanisms underlying this activity, we investigated key regulatory molecules involved in this pathway.

**Figure 8:**
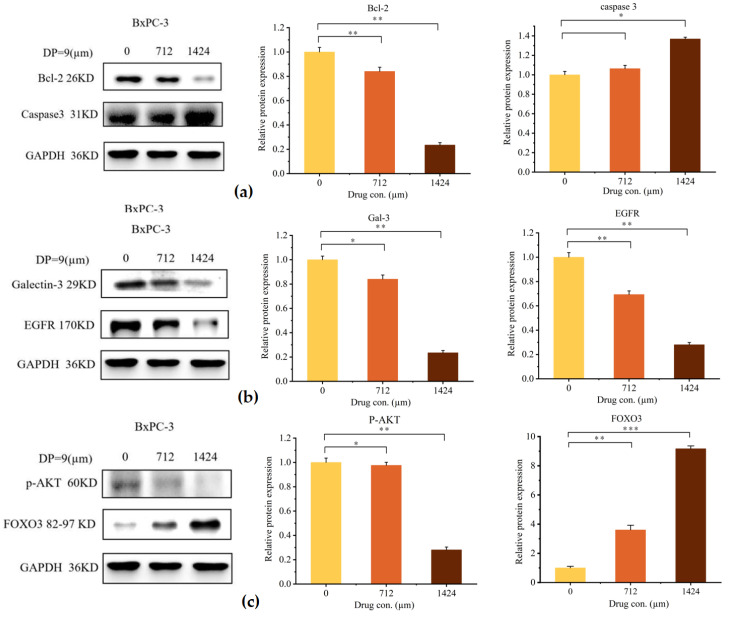
Anti-pancreatic cancer (BxPC-3 cell) activity of DP9 in detecting the expression level of various factors. (**a**) The effects of DP9 on the expression of Bcl-2 and Caspase3. (**b**) The effects of DP9 on the expression of Gal-3 and EGFR. (**c**) The effects of DP9 on the expression of p-AKT and FOXO3. Data presented as average ± standard deviation (*n* = 3, * *p* < 0.05, ** *p* < 0.01, *** *p* < 0.001 compared with 0 µM).

Western blot analysis revealed that DP9 treatment significantly downregulated Gal-3 protein expression after 48 h of exposure. Furthermore, we examined the expression of apoptosis-related proteins Bcl-2 and Caspase-3 following 48 h of DP9 treatment. As shown in Figure 8a–c, DP9 treatment dose-dependently decreases Bcl-2 levels while simultaneously increasing cleaved Caspase-3 protein levels. This activation of Caspase-3, the critical terminal executioner protease in the apoptotic cascade, indicates DP9’s pro-apoptotic effect. Notably, DP9 treatment also significantly upregulated FOXO3 protein levels. As a member of the O subclass of forkhead transcription factors, characterized by its distinctive forkhead DNA-binding domain, FOXO3 plays a pivotal role in regulating cell cycle arrest and apoptosis.

The observed experimental phenomena align with previous research, demonstrating that DP9 significantly slows cell cycle progression [40], induces apoptosis [41], and inhibits angiogenesis [42]. These findings suggest that DP9 may enhance FOXO3 expression, initiating a cascade of biological processes that contribute to its anti-pancreatic cancer effects. Further investigation, as depicted in Figure 8a–c, reveals that DP9 treatment decreases the expression of EGFR, phosphorylated AKT, and Gal-3 in a dose-dependent manner while increasing FOXO3 levels.

## 3. Discussion

**Figure 9:**
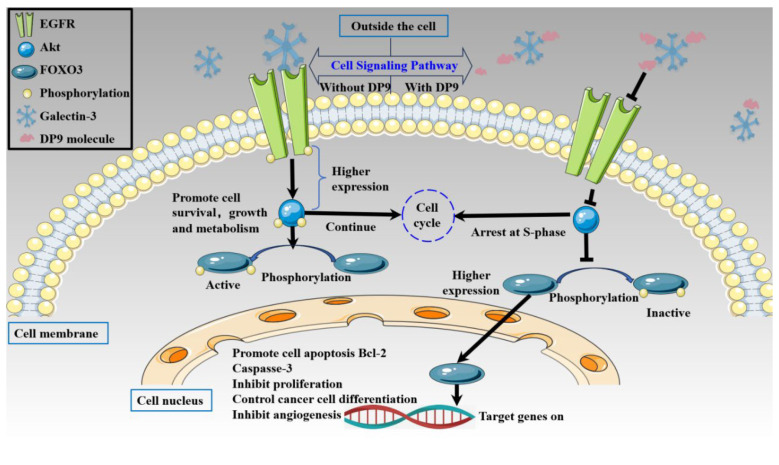
Schematic showing the proposed mechanism of DP9 in the BxPC-3 cell targeting the Gal-3/EGFR/AKT/FOXO3 signaling pathway. The blue symbol with a P in the circle denotes phosphorylation.

DP9, an oligosaccharide isolated from *G. lemaneiformis* polysaccharides via acid hydrolysis, was identified and validated in this study. DP9 effectively disrupts the interaction between EGFR and Gal-3, and suppresses the Gal-3/EGFR/AKT/FOXO3 signaling pathway, thereby exerting anti-pancreatic cancer activity. The proposed molecular mechanism, illustrated in Figure 9, demonstrates how DP9 binds to Gal-3, impedes EGFR–Gal-3 complex formation, and inhibits the signaling cascade, culminating in potent in vitro anti-pancreatic cytotoxic effects.

This study focused on EGFR, a crucial Gal-3 ligand implicated in tumor development. The structural characterization of DP9 reveals its relatively low molecular weight, which may contribute to its enhanced bioavailability compared to that of larger polysaccharide-based Gal-3 inhibitors reported previously [20,21,43]. DP9’s lower molecular weight, simpler chemical structure, and higher water solubility result in predictable pharmacokinetics and pharmacodynamics, leading to simpler dosing regimens for patients. As a signal transduction inhibitor, DP9 binds directly to targeted proteins and intracellular targets, effectively blocking essential signal transduction pathways required for tumor growth and proliferation.

In summary, DP9 exhibits potent in vitro anti-cancer activity against human pancreatic cancer cells, with low toxicity observed in normal pancreatic ductal epithelial cells. Mechanistic studies reveal that DP9 inhibits cell proliferation, promotes apoptosis, induces cell cycle arrest, and impedes migration, invasion, and angiogenesis by blocking the Gal-3/EGFR/AKT/FOXO3 signaling pathway. Furthermore, DP9 significantly upregulates the transcription factor FOXO3 in a concentration-dependent manner, leading to the decreased expression of Gal-3 and EGFR. The binding mode of DP9 toward Gal-3 interferes with EGFR-Gal-3 interactions, exerting relevant biological activities. High extracellular concentrations of DP9 may serve as a significant competitive inhibitor of EGFR for Gal-3. Consequently, the Gal-3 antagonist DP9 holds promise as a novel anti-cancer drug candidate.

## 4. Materials and Methods

### 4.1. Materials and Reagents

#### 4.1.1. General Reagents

The seaweed *G. lemaneiformis* was purchased from Nan’ao Island, China, where samples were dried and ground into a powder prior to polysaccharide extraction. Purified agaro-oligosaccharide standards with defined degrees of polymerization, including agaro-triose (AG3), agaro-pentose (AG5), agaro-heptose (AG7) and agaro-nonose (AG9), as well as neoagaro-biose (NA2), neoagaro-tetraose (NA4), neoagaro-hexaose (NA6), neoagaro-octaose (NA8), neoagaro-decaose (NA10) and neoagaro-dodecaose (NA12), were commercially obtained from Qingdao HEHAI Biotech Co., Ltd. (Qingdao, China), with these reference standards originating from Gelidium amansii. For hemagglutination assays, neoagaro-oligosaccharides (DP2, DP4, DP6, DP8, and DP10) with an even-numbered degree of polymerization were prepared in our laboratory through the enzymatic hydrolysis of *G. lemaneiformis* crude polysaccharides using an agarolytic bacterial strain, ZC1T, exhibiting β-galactosidase activity, kindly provided by Prof. Hu Zhong’s laboratory [44]. Recombinant human Gal-3 was purchased from ACRO Biosystems (Beijing, China), while bovine serum albumin and N-acetyl-D-lactosamine (LacNAc) were acquired from Aladdin (Shanghai, China) and Baishun Biotechnology (Shanghai, China), respectively. Matrigel with reduced growth factors was obtained from BD Biosciences (Paramus, NJ, USA), and all antibodies used in the experiments are detailed in Supplementary Table S1. Additional essential reagents included an SDS-PAGE gel preparation kit, a cell cycle and apoptosis analysis kit, a Methyl Thiazolyl Tetrazolium (MTT) cell proliferation and cytotoxicity assay kit, and an ultra-hypersensitive enhanced chemiluminescence (ECL) kit from Beyotime Biotechnology Co., Ltd. (Beijing, China), along with chromatographic-grade acetonitrile and other chemical reagents from Xilong Chemical Co., Ltd. (Shantou, China).

#### 4.1.2. Cell Lines and Cell Culture

The human pancreatic cancer cell line BxPC-3 and its specialized culture medium (CM-0042) were obtained from Procell Life Science & Technology Co., Ltd. (Wuhan, China). Cells were maintained at 37 °C in a humidified incubator with 5% CO_2_ under standard culture conditions.

### 4.2. Determination of DP9 Oligosaccharide Structural Characterization

#### 4.2.1. Extraction of Crude Polysaccharides from *G. lemaneiformis*

A 10 g sample of *G. lemaneiformis* powder was mixed with 500 mL of distilled water and subjected to extraction using an ultrasonic-microwave apparatus (CW-2000, Shanghai Xintuo Co., Shanghai, China) for 30 min. The extraction was performed at a microwave power of 500 W and an ultrasonic power of 50 W. After extraction, the mixture was filtered through gauze, and the supernatant was collected. Subsequently, a three-fold volume of anhydrous ethanol was added to the supernatant to precipitate the polysaccharides. The solution was then kept overnight at 4 °C. Finally, the precipitate was separated by centrifugation (4000 r/min, 10 min) and freeze-dried to obtain the crude polysaccharides from *G. lemaneiformis*.

#### 4.2.2. Acidolysis of *G. lemaneiformis* Crude Polysaccharides

One gram of crude polysaccharides from *G. lemaneiformis* was dissolved in one hundred milliliters of distilled water under heating until complete dissolution. Then, 0.5 mL of 2 mol/L sulfuric acid was added, and the mixture was stirred and hydrolyzed in an 80 °C water bath for 240 min. After hydrolysis, the solution was rapidly cooled to room temperature using an ice-water bath. The cooled hydrolysate was neutralized with BaCO_3_ and centrifuged (4000 r/min, 10 min). The resulting supernatant was concentrated to 10 mL via rotary evaporation at 70 °C. A four-fold volume of anhydrous ethanol was then added to the concentrated solution to precipitate residual polysaccharides, followed by centrifugation (4000 r/min, 5 min) to remove the precipitate. The supernatant was further concentrated by rotary evaporation at 70 °C to obtain the crude oligosaccharide mixture. Finally, the oligosaccharide mixture was lyophilized to yield a dry product.

#### 4.2.3. High-Performance Liquid Chromatography-Evaporative Light-Scattering Detector (HPLC-ELSD) Analysis of Acid Hydrolysis Products

The compositional analysis was conducted using an NH2P-50 4E column (150 × 4 mm; Shodex Asahipak, Tokyo, Japan). A mixed standard solution containing pure AG3, AG5, and AG7 (each at 5 mg/mL) and AG9 (4 mg/mL) was prepared by equal volume mixing to identify oligosaccharides with different degrees of polymerization under identical analytical conditions. The mobile phase employed a gradient elution program: 67% to 50% acetonitrile from 0 to 7 min, maintained at 50% acetonitrile from 7 to 20 min, with a constant flow rate of 0.5 mL/min. Detection was performed using an Evaporative Light Scattering Detector (ELSD6100, ALLCHROM, Tianjin, China), with component identification achieved by comparing retention times with the reference standards.

#### 4.2.4. Separation of Oligosaccharides from *G. lemaneiformis*

Oligosaccharide separation was performed using a Bio-Gel P-2 (Bio-Rad, Hercules, CA, USA) packed chromatographic column (XK 16/100, Cytiva, Fairfield, CT, USA). A 2 mL aliquot of the concentrated oligosaccharide mixture was loaded onto the column at a flow rate of 0.2 mL/min, with deionized water serving as the mobile phase. Fractions were collected automatically every 3 mL (15 min intervals) using a fraction collector. Elution profiles were monitored in real-time using a refractive index detector (SFD RI2000, Erlangen, Germany). The collected fractions were pooled according to their degree of polymerization and subsequently lyophilized to obtain purified *G. lemaneiformis* oligosaccharides (DP3, DP5, DP7, DP9, and DP11). The identity and purity of each oligosaccharide fraction were verified by HPLC-ELSD analysis.

#### 4.2.5. Thin-Layer Chromatography (TLC) Analysis of Oligosaccharides

The hydrolysis products (DP3-DP11) were analyzed by TLC using pure NA2, NA4, NA6, NA8, and NA10 as reference standards. Samples were spotted onto a Merck Silica Gel 60 TLC plate (20 cm length), approximately 15 mm from the bottom edge. Chromatographic separation was performed in a glass beaker using a mobile phase consisting of n-butanol: glacial acetic acid: water (2:1:1, *v*/*v*) for approximately 4.5 h. After development, the TLC plate was sprayed with a visualization reagent (10% sulfuric acid in deionized water, *v*/*v*) and dried at 85 °C for 15 min to reveal the oligosaccharide spots.

#### 4.2.6. Fluorophore-Assisted Carbohydrate Electrophoresis (Face) Analysis of Oligosaccharides

The dried oligosaccharide products were derivatized with 8-aminonaphthalene-1,3,6-trisulfonic acid (ANTS) according to the method described by Cheong et al. [45]. Briefly, 50 µL of 0.1 mol/L ANTS solution (in 15% *v*/*v* aqueous acetic acid) and 50 µL of 1 mol/L sodium cyanoborohydride (in dimethyl sulfoxide, DMSO) were mixed with the dried samples and allowed to react at 37 °C for 17 h. The reaction mixture was then dried under a stream of nitrogen gas.

The ANTS-derivatized samples were subsequently redissolved in 0.5 mL of 6 mol/L urea solution. Electrophoresis was performed using a vertical slab gel apparatus with 10 cm glass plates, 1.0 mm spacers, and 0.3 cm wide wells. The gel system consisted of a 32% (*w*/*v*) polyacrylamide resolving gel and an 8% (*w*/*v*) polyacrylamide stacking gel. Electrophoresis was carried out in 0.1 mol/L Tris-boric acid buffer (pH 8.2) at a constant voltage of 300 V for 60 min, with cooling in an ice bath. The run was terminated when the bromophenol blue tracking dye migrated to the predetermined position. The fluorescently labeled oligosaccharides were visualized using a Gel Doc2000TM Gel Imaging System (Bio-Rad, Hercules, CA, USA) under 365 nm UV illumination.

#### 4.2.7. Analysis of Monosaccharides and Their Ratios (Anhydro-Galactose/Galactose, Angal/Gal) in Dp9

To analyze the monosaccharide composition of DP9 and identify the main types of monosaccharides it contained, modified acid hydrolyzation of DP9 and HPLC determination was applied [46], which is described in detail in S3 of the Supplementary Material.

Based on the above results, to further measure the AnGal/Gal molar ratios of DP9, two-step acid hydrolysis and gas chromatography–mass spectrometry (GC-MS) analysis were applied [13]. Hydrolysis and derivatization were carried out. A two-step acid hydrolysis strategy could retain the AnGal structure of DP9 and polysaccharides from *G. lemaneiformis*. For the first step of hydrolysis, 100 μL of 1 mg/mL DP9 or polysaccharide solution was added to 50 μL of 80 mg/mL methylmorpholine–borane complex (MMB) and trifluoroacetic acid (TFA) at a final concentration of 2.5 M and then hydrolyzed at 80 °C for 30 min. For the second-step complete hydrolysis of residual polysaccharide, we added 1.5 M TFA after residue reconstitution in deionized water. Then, this was hydrolyzed in a 120 °C oil bath for 1 h. The hydrolysis products were derivatized with acetic anhydride to obtain acetates, which were used for GC-MS analysis.

#### 4.2.8. Fourier-Transform Infrared (FTIR) Analysis of DP9

FTIR spectroscopy was performed using a Nicolet iS50 spectrometer (Thermo Fisher Scientific, Waltham, MA, USA). The tested samples were prepared by homogenizing 1 mg of DP9 with 100 mg of potassium bromide (KBr) and were pressed into pellets. Spectra were acquired in the mid-infrared region (4000–400 cm^−1^) at a resolution of 4 cm^−1^.

#### 4.2.9. Electrospray Ionization Mass Spectrometry (ESI-MS) Analysis of DP9

The oligosaccharide fractions were analyzed by ESI-MS using a VG Platform single-quadrupole mass spectrometer (Micromass, Altrincham, UK) at Shantou University’s Instrumentation Center (Shantou, China). The instruments used are described in detail in Supplementary Material S8.

#### 4.2.10. Nuclear Magnetic Resonance (NMR) Analysis of DP9

The NMR analysis of DP9 was performed by first dissolving 20 mg of the freeze-dried sample in 0.5 mL deuterium oxide (D2O). All NMR spectra were acquired on a Bruker AVANCE HD III 600 MHz spectrometer, including one-dimensional ^1^H, ^13^C, and DEPT-135 spectra along with two-dimensional COSY, HSQC, HMBC, and NOESY experiments. Data processing and analysis were conducted using MestReNova vl4.0.0-23239 software, with chemical shifts referenced in parts per million (ppm) using residual HDO (δ_H_ 4.70 ppm) as the internal standard for proton NMR and tetramethylsilane (TMS; δ_C_ 0.00 ppm) for carbon NMR measurements.

### 4.3. Computational Analysis of Molecular Interactions Between DP9 and Human Gal-3

#### 4.3.1. Molecular Docking

We proposed the major and minor DP9 chemical structures in Table 2 and Supplementary Table S2. We conducted MM2 calculation to minimize energy. Human galectin-3 protein (PDB id: 1a3k) was obtained from the RCSB database (https://www.pdbus.org, accessed on 24 November 2024), and we cleaned surrounding water molecules on Pymol (Version 3.0 Schrödinger, LLC, New York, NY, USA). For investigating all possible bindings using AutoDock Vina tool 12.0 [47], a blind grid box was set up to cover the whole protein molecule based on center (6, 0, −8) and size (40, 35, 40). Due to the limitations of the heavy atom number investigated in Antodock Vina tool, DP9(G-L6S) 1 to 6 and DP9(G-LA) were proposed on LeDock (https://www.lephar.com/software, accessed on 24 November 2024) in triplicate. Docked results were visualized by UCSF Chimera 1.15 [48].

#### 4.3.2. Molecular Dynamic Simulation

Molecular dynamics simulations were performed using the following protocol: The DP9 structure was first hydrogenated using GaussView Version 5.0 and converted into topological format using Sobtop (http://sobereva.com/soft/Sobtop, accessed on 16 July 2024). The ligand-free and water-free Gal-3 protein was positioned at the center of a cubic simulation box. The system was solvated with 9563 TIP3P water molecules and neutralized by adding four chloride ions, maintaining a total of 9563 solvent molecules in the electroneutral system. Force field parameters were assigned as follows: the GAFF force field for DP9 and the AMBER ff99SB-ILDN force field for Gal-3 [49]. All simulations were conducted using the GROMACS 2018.6 package [37] under isothermal–isobaric conditions at 289.15 K and 1 bar pressure, with a 2 fs integration time step. The temperature was maintained constant at 289.15 K throughout the simulation. Protein residue fluctuations, analyzed based on B-factor values, were visualized using color mapping in PyMOL Version 3.0.3. For comparison, the known Gal-3 inhibitor LacNAc [37] was selected to serve as a positive control.

### 4.4. Gal-3 Inhibitory Effect and In Vitro Anti-Pancreatic Cancer Activity of DP9

#### 4.4.1. Gal-3 Inhibitory Effect of DP9 Through Hemagglutination Assay

The Gal-3 inhibitory potential of DP9 was initially evaluated using a chicken erythrocyte hemagglutination assay, following the established protocol of [50]. This assay systematically compared the inhibitory effects across the complete series of oligosaccharide hydrolysis products (DP3–DP11) to identify the most potent Gal-3 inhibitor. The detailed experimental methodology is comprehensively described in Supplementary Material S2.

#### 4.4.2. Effect of DP9 on BxPC-3 Cell Proliferation

BxPC-3 human pancreatic cancer cells were maintained in Dulbecco’s modified eagle medium (DMEM, Procell, Wuhan, China) supplemented with 10% (*v*/*v*) heat-inactivated fetal bovine serum (FBS), 2.0 mmol/L glutamine, 100 U/mL penicillin, and 100 μg/mL streptomycin. Cells were cultured at 37 °C in a humidified atmosphere containing 5% CO_2_. For experiments, cells in the logarithmic growth phase were harvested by gentle scraping and resuspended in fresh DMEM.

Cell proliferation was assessed using the MTT assay. Briefly, cell suspensions (1 × 10^5^ cells/mL) were seeded in 96-well flat-bottom plates (100 μL/well) and allowed to adhere for 24 h. After removing the culture supernatant, cells were treated with DP9 at concentrations of 356, 712, and 1424 μM for either 24 or 48 h. Following treatment, 20 μL of MTT solution (5 mg/mL in phosphate-buffer saline, PBS) was added to each well and incubated for 4 h in the dark. The formazan crystals formed were dissolved in 200 μL DMSO per well after careful removal of the medium. Absorbance was measured at 570 nm using a microplate reader, with cell viability expressed as the percentage of absorbance relative to the untreated controls.

#### 4.4.3. Effect of DP9 on BxPC-3 Cell Migration

Cell migration was assessed using a Transwell assay with 8 μm pore polycarbonate membranes (Corning, NY, USA). BxPC-3 cells (1.5 × 10^5^ cells/well) suspended in 100 μL serum-free medium containing DP9 (0, 712, or 1424 μM) were seeded in the upper chamber, while the lower chamber contained medium supplemented with 15% FBS as a chemoattractant. After 8 h incubation at 37 °C, non-migrated cells on the upper membrane surface were removed using a cotton swab, and migrated cells on the lower surface were fixed and stained with 0.1% crystal violet. Cell migration was quantified by counting five random fields per well under an Olympus BX51 microscope. The migration rate calculation formula is as follows: migration rate (%) = (number of migrated cells/number of migrated cells in control group) × 100%; the wound healing rate calculation formula is as follows: healing rate (%) = (scratch area at 0 h—scratch area at specific time point)/scratch area at 0 h × 100%. The invasion assay methodology is detailed in Supplemental Material S6.

#### 4.4.4. Effect of DP9 on BxPC-3 Cell Cycle

For cell cycle analysis, DP9-treated BxPC-3 cells were harvested by trypsinization after 24 h of exposure, washed with PBS, and fixed in 75% ethanol at −20 °C overnight. Fixed cells were then resuspended in 500 μL PBS containing RNase A (0.1 mg/mL) and stained with propidium iodide for 30 min at room temperature in the dark. DNA content was analyzed using a BD flow cytometer equipped with CellQuest Pro software (BD Biosciences, Milpitas, CA, USA), and cell cycle distribution was determined using ModFit LT Version 3.0 software. Additional data on DP9 cytotoxicity in L929 cells (Supplemental Material S5) and DP9-induced apoptosis (Supplemental Material S7) are provided in the Supplementary Information.

#### 4.4.5. Effect of DP9 on Scratch Wound Assay

BxPC-3 cells in the logarithmic growth phase were seeded in 12-well plates at a density of 6 × 10^5^ cells/mL and allowed to adhere for 24 h in a humidified incubator (37 °C, 5% CO_2_). When cells reached 80–90% confluency, two parallel linear wounds were created in each well using a 200 μL pipette tip. The wells were then gently washed twice with 500 μL PBS to remove dislodged cells. After PBS removal, cells were treated with serum-free medium containing either 0 (control), 712, or 1424 μM DP9, with at least three replicate wells per treatment group. Wound closure was monitored and documented at 0, 12 and 24 h post-treatment using phase-contrast microscopy.

#### 4.4.6. Effect of DP9 on Angiogenesis

The in vitro angiogenesis potential was evaluated using a Matrigel-based tube formation assay. Briefly, 96-well plates were coated with 50 μL of chilled Matrigel per well and allowed to polymerize at 37 °C for 30 min. BxPC-3 cells (1 × 10^5^ cells/well) suspended in medium containing varying concentrations of DP9 (0, 712, or 1424 μM) were then seeded onto the Matrigel surface. Following 12 h of incubation at 37 °C under 5% CO_2_, capillary-like network formation was assessed and imaged using an Olympus IX51 inverted microscope (Tokyo, Japan). Three independent experiments were performed with quintuplicate wells for each condition.

#### 4.4.7. Effect of DP9 on Western Blot Analysis

For protein expression analysis, BxPC-3 cells under different treatments were lysed in RIPA buffer containing the inhibitors on ice for 30 min. Protein concentrations were determined by the BCA assay, and equal amounts (10 μL per lane) were separated on different-concentration SDS-PAGE gels (5% gel and 80 V for stacking, 10% gel and 150 V for separation). Proteins were transferred to PVDF membranes using semi-dry transfer (300 mA, 90 min), blocked with 5% non-fat milk for 2 h, and incubated with primary antibodies (Supplementary Table S1) at 4 °C overnight. After three PBST washes, membranes were incubated with secondary antibodies for 1 h at room temperature. Signals were developed with ECL substrate and quantified using ImageJ (NIH) (1.53 k) after normalization to β-actin.

### 4.5. Statistical Analysis

All experiments were conducted with a minimum of three biological replicates to ensure reproducibility. Quantitative data are expressed as the mean ± standard deviation (SD). Statistical significance was determined using one-way analysis of variance (ANOVA) followed by appropriate post hoc tests, with *p*-values < 0.05 considered statistically significant. All analyses were performed using SPSS Statistics software (Version 20; IBM Corporation, Ammonk, NY, USA).

## 5. Conclusions

The study’s findings establish DP9 as a highly promising Gal-3 inhibitor derived from a natural, non-toxic source. Its unique structural features and potent biological activities position it as a strong candidate for the development of novel therapeutic agents. Specifically, DP9 exhibits robust anti-pancreatic cancer properties, effectively suppressing cell proliferation, inducing apoptosis, arresting the cell cycle at the S phase, and inhibiting angiogenesis and tumor cell migration and invasion. Furthermore, DP9’s ability to modulate the Gal-3/EGFR/AKT/FOXO3 signaling pathway underscores its therapeutic potential. Importantly, its natural origin and lack of toxicity make DP9 an exceptionally attractive candidate for advancing treatments targeting pancreatic cancer and other Gal-3-mediated diseases.

## Figures and Tables

**Table 1:** Assignment of ^1^H and ^13^C chemical shifts for each sugar residue in DP9 from G. lemaneiformis.

Residue		Chemical Shifts δ (ppm)							
		Type	1	2	3	4	5	6	-OMe
G	→3)-β-D-Galp-(1→	H	4.51	3.53	3.72	4.04	3.65	3.66/3.72	
		C	101.84	69.74	81.70	68.18	75.03	60.92	
LA	→4)-α-L-AnGalp-(1→	H	5.06	4.05	4.46	4.57	4.48	3.93/4.15	
		C	98.06	69.11	79.63	76.89	74.91	68.72	
G′	→3)-β-D-Galp-(1→	H	4.37	3.63	3.68	4.04	3.65	3.66/3.72	
	G→L6S	C	102.86	70.77	80.28	68.18	75.03	60.92	
L6S	→4)-α-L-Galp6S-(1→	H	5.21	3.79	3.87	4.20	4.30	4.21	
		C	100.80	68.96	68.57	77.92	69.49	67.01	
G6M	→3)-β-D-Galp-6-OMe-(1→	H	4.32	3.52	3.72	4.04	3.78	3.60	3.33
		C	103.15	70.92	81.70	68.18	73.26	71.45	58.57
Gnr	β-D-Galp-(1→	H	4.46	3.40	3.56	3.84	3.65	3.66/3.72	
		C	102.12	70.38	72.48	68.57	75.03	60.92	
Grα	→3)-α-D-Galp	H	5.18	3.88	3.89	4.10	3.71	3.66/3.72	
		C	92.37	68.57	78.85	68.72	69.49	60.92	
Grβ	→3)-β-D-Galp	H	4.52	3.59	3.69	4.01	3.70	3.66/3.72	
		C	96.15	71.31	82.09	69.88	74.94	60.92	

## Data Availability

Data is contained within the article and Supplementary Materials.

## Supplementary Materials

The following supporting information can be downloaded at: https://www.mdpi.com/article/10.3390/md23110423/s1, Figure S1: The hemagglutination assay test plate for detection of the oligosaccharide anticoagulation ability; Figure S2: The monosaccharide chromatogram of DP9; Figure S3: Cytotoxicity and proliferation effects of DP9 for L929 cells; Figure S4: Effect of DP9 on BxPC-3 cell invasion (Transwell assay with Matrigel coating). The number of invasive cells was significantly reduced in a dose-dependent manner with DP9 treatment, compared to the control group; Figure S5: Effect of DP9 on apoptosis of BxPC-3 cells and the number of apoptotic BxPC-3 cells under different concentrations of DP9; Table S1: Antibodies used for Western blotting; Table S2: Chemical structure of DP9(GLA), DP9(G6MLA)1-DP9(G6MLA)6; Table S3: AutoDock Vina results docked to 1A3K.
