# Supplementary material for: Novel Sulfated Oligosaccharide DP9 from Marine Algae, Gracilaria lemaneiformis: A Potent Galectin-3 Inhibitor for Pancreatic Cancer Therapy
Source: Mar Drugs. 2025 Oct 30;23(11):423. doi: 10.3390/md23110423 (PMC12653795; doi:10.3390/md23110423)
Supplement: Supplementary file 1 [file marinedrugs-23-00423-s001.zip › marinedrugs-3940062-supplementary.pdf]

Supplementary material

Novel Sulfated Oligosaccharide DP9 from *Gracilaria lemaneiformis*: A Potent Galectin-3 Inhibitor for Pancreatic Cancer Therapy

Zhicong Liu†, Pingting Liu†, Fengyuan Li†, Yang Liu*

Guangdong Provincial Key Laboratory of Marine Biotechnology, Guangdong Engineering Technology Research Center of Offshore Environmental Pollution Control, Department of Biology, College of Science, Shantou University, Shantou, Guangdong 515063, P.R. China.

S1. Antibodies used in experiments

Table S-1 Antibodies used for Western blotting

Antibody	Company	Catalog
EGFR Rabbit Polyclonal Antibody (KO Validated)	Beyotime Biotechnology	AF5153
FoxO3a	Beyotime Biotechnology	AF609
p-Akt (Ser473)	Beyotime Biotechnology	AA329
Rabbit Anti-Galectin 3 antibody	Bioss Biotechnology	Bs-20699R
GAPDH	Beyotime Biotechnology	AF0006
Caspase-3rabbit polyclonal antibody	Beyotime Biotechnology	AF0081
Bcl-2 Rabbit Polyclonal Antibody	Beyotime Biotechnology	AFO060
HRP-labeled Goat Anti-Rabbit IgG (H+L)	Beyotime Biotechnology	A0208

S2. Galectin-3 (Gal-3)-mediated hemagglutination assay

Hemagglutination assay was performed as following steps. Firstly, mixed 25 μL of oligosaccharides in different concentrations and different degrees of polymerization (dissolved in PBS, pH 7.4) with 25 μL of Gal-3 (2 $\mu mol \cdot L^{-1}$) in a V-shaped transparent 96-well plate for 10 min. Secondly, 25 μL of 1% (w/v) BSA solution (dissolved in PBS, pH 7.4) was added into 50 μL of oligosaccharide sample and Gal-3 mixture within the sample well. Finally, 25 μL of 4% (v/v) chicken erythrocyte suspension was then added into the sample well. Here, the positive control (PC) was set including PBS instead of oligosaccharide sample, Gal-3 (2 $\mu mol \cdot L^{-1}$), 1% (w/v) BSA and 4% (v/v) chicken erythrocyte with 25 μL each. The negative control (NC) was set including 50 μL PBS without Gal-3, 1% (w/v) BSA and 4% (v/v) chicken erythrocyte with 25 μL each. For comparison, N-acetyl-D-lactosamine (LacNAc) was used as a positive drug instead of oligosaccharide sample. When the sample mixture reacted at 25 °C for 0.5 h, the morphology of chicken erythrocytes was observed. If chicken erythrocytes naturally settled into a small dot, it indicated that there was no agglutination. If chicken erythrocytes agglomerated in large numbers around the well wall, it indicated that there was complete agglutination. The inhibitory activity of oligosaccharide was expressed as the minimum concentration (MIC) of the oligosaccharides required to completely inhibit erythrocytes agglutination.

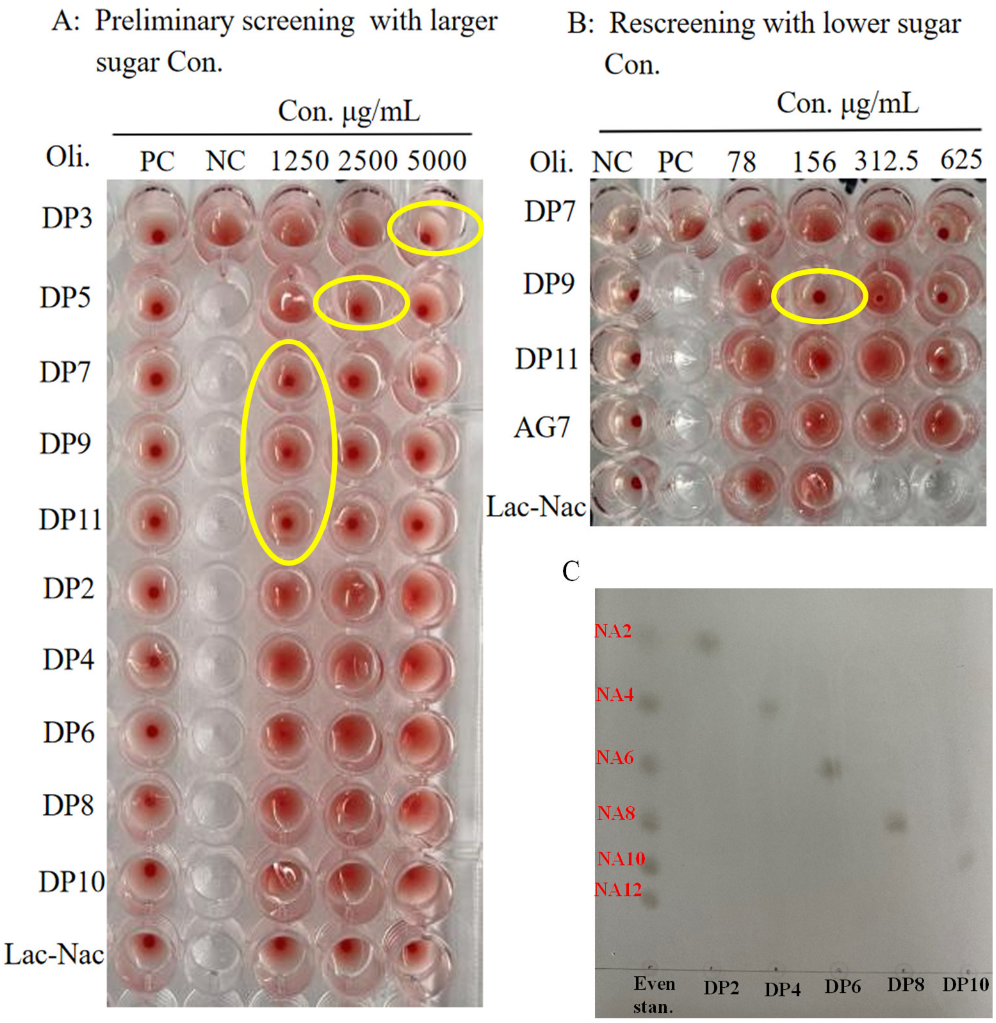

Figure S-1. The hemagglutination assay test plate for detection of the oligosaccharide anticoagulation ability. (A) Preliminary screening of anticoagulation ability of the isolated oligosaccharides with high sugar concentration. (B) The hemagglutination test plate of oligosaccharides with stronger anticoagulant ability used lower sugar concentration. The yellow circle indicates MIC of various oligosaccharides. (C) The TLC results of various oligosaccharides (DP2, DP4, DP6, DP 8, and DP 10) with even-numbers degree of polymerization from G. lemaneiformis.

In the screening experiments, it was found that DP3 and DP5 had weak anticoagulation abilities, while DP7, DP9 and DP11 had certain anticoagulation abilities. Therefore, DP7, DP9, and DP11 were selected for rescreening at lower sugar concentrations (showed in Figure S-1.B). MIC of DP7, DP9 and DP11 were 625, 156 and 1250 μg/mL respectively, while purchased AG7 with the same polymerizing degree of DP7 did not show any inhibitory ability. DP11 and AG7 had parity inhibitory ability

at the concentration of 625 μg/mL. Positive drug LacNAc had parity inhibitory ability at 156 μg/mL. The above experiments indicated DP9 had the strongest inhibitory ability and the strongest Gal-3 affinity. The MIC of oligosaccharides from low to high were sequentially arranged as DP9, DP7, DP11, DP5, and DP3, thus the order of oligosaccharides with affinity to Gal-3 from strong to weak was DP9, DP7, DP11, DP5, and DP3.

Additionally, the TLC results of various oligosaccharides (DP2, DP4, DP6, DP 8, and DP 10) obtained through enzymatic hydrolysis from *G. lemaneiformis* was displayed in Figure S-1 C.

S3. The monosaccharide chromatogram of DP9.

5mg of DP9 was hydrolyzed with 1mL of 2mol/L trifluoroacetic acid (TFA) at 120°C for 4h in a sealed reaction tube. Excess TFA was removed under a nitrogen stream, and the residue was reconstituted in 1mL of distilled water. For derivatization, 100 μL of the hydrolysate was reacted with 100 μL of 0.6 mol/L NaOH and 200μL of 0.5mol/L 1-phenyl-3-methyl-5-pyrazolone (PMP) at 70°C for 2h. The resulting PMP derivatives were extracted with dichloromethane prior to HPLC analysis. HPLC separation was performed using a Hypersil ODS2 column (5μm, 250mm×4.6mm) with a mobile phase consisting of 0.1mol/L phosphate buffer (pH 6.8) and acetonitrile (84:16, v/v) at a flow rate of 0.8mL/min at 30°C, with detection at 254nm.

As Figure S-2 shown, by comparing the retention times between standard monosaccharides and acid-hydrolyzed DP9, galactose was revealed as the primary component of DP9. This was attributed to the acid-induced conversion of 3,6-anhydro-

galactose (AnGal) to galactose (Gal) during hydrolysis, explaining the exclusive detection of galactose in the chromatographic profile.

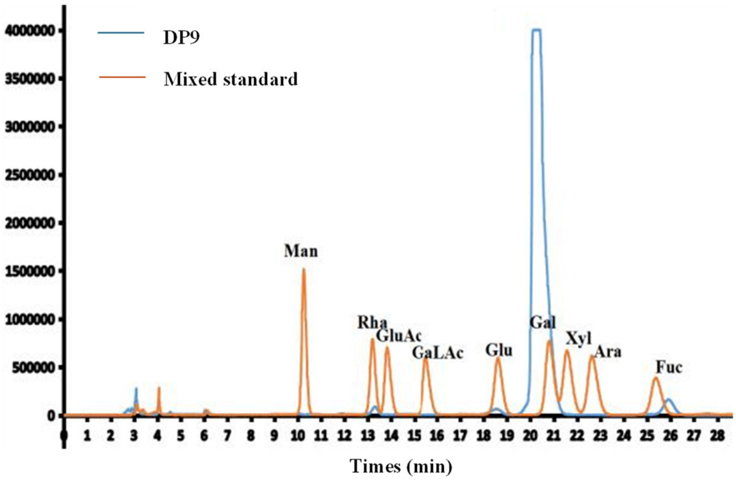

Figure S-2. The monosaccharide chromatogram of DP9.

S4. Molecular docking results

Based on major and minor repeating units analyzed in Fig. 5, DP9(G-LA), six DP9(G6M-LA)s and six DP9(G-L6S)s were proposed and drawn as respective major and minor chemical structures.

Table S-2. Chemical structure of DP9(GLA), DP9(G6MLA)1-DP9(G6MLA)6.

Name	Chemical Structure
DP9(G-LA)	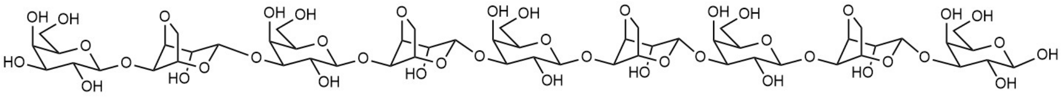
DP9(G6M-LA)1	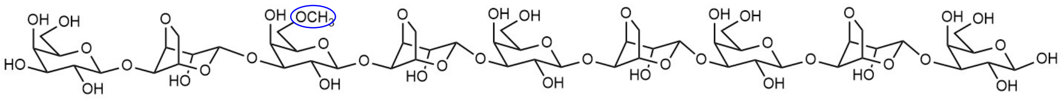
DP9(G6M-LA)2	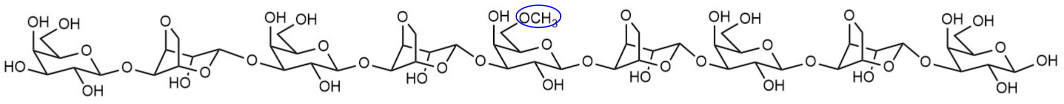
DP9(G6M-LA)3	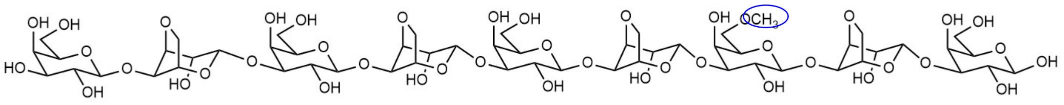

DP9(G6M-LA)4	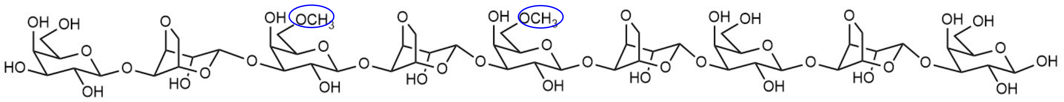
DP9(G6M-LA)5	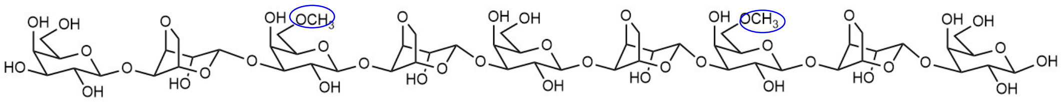
DP9(G6M-LA)6	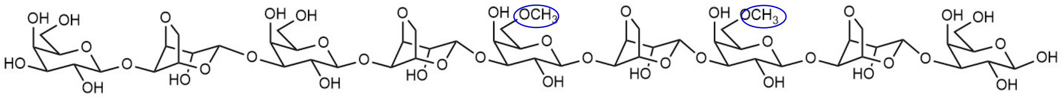

DP9(G6M-LA)

Six DP9(G6MLA) molecules were predicted and docked. DP9(G6MLA)1-DP9(G6MLA)6 showed similar binding scores instead of DP9(G6MLA)2, which had highest binding score and lowest binding affinities in DP9-Gal3 interactions.

Table S-3. AutoDock Vina results docked to 1A3K.

Ligand Name	AutoDock Vina Score (kcal/mol)
DP9(G6M-LA)1	-6.5
DP9(G6M-LA)2	-6.1
DP9(G6M-LA)3	-6.5
DP9(G6M-LA)4	-6.4
DP9(G6M-LA)5	-6.5
DP9(G6M-LA)6	-6.6
DP9(G-LA)	-6.7
LacNAc	-5.3

S5. Cytotoxicity of DP9 on L929 cells

Logarithmic growth L929 cells were digested with 0.25% trypsin and terminated digestion using DMEM medium within 10% FBS. Cell suspension was centrifuged at 1000 r/min for 5 min before discarding the supernatant. 100 μL cells were seeded at

$1{\times}10^4$ cell/well in the 96-well plate in 5% CO2 incubator at 37°C. Removed original medium after 24 h. Experimental groups treated with 100 μL fresh medium at 356 μM, 712 μM and 1424 μM DP9. Control group added equal volume of normal medium. Six replicates for each group and treated for another 24 h. After drug intervention, 10 μL CCK-8 reagent and 90 μL fresh medium were added to each well. Measured absorbance (OD) at 450 nm.

No obvious cytotoxicity on L929 cells was observed in Figure S-3. Viability of Normal control (NC) and DP9 at concentrations of 356 μM, 712 μM, and 1424 μM were 100%, 95.03%, 99.77%, and 100.13%, respectively. No significant difference in cell survival rate among different DP9 concentrations compared with NC group, and no dose dependence on experimental groups.

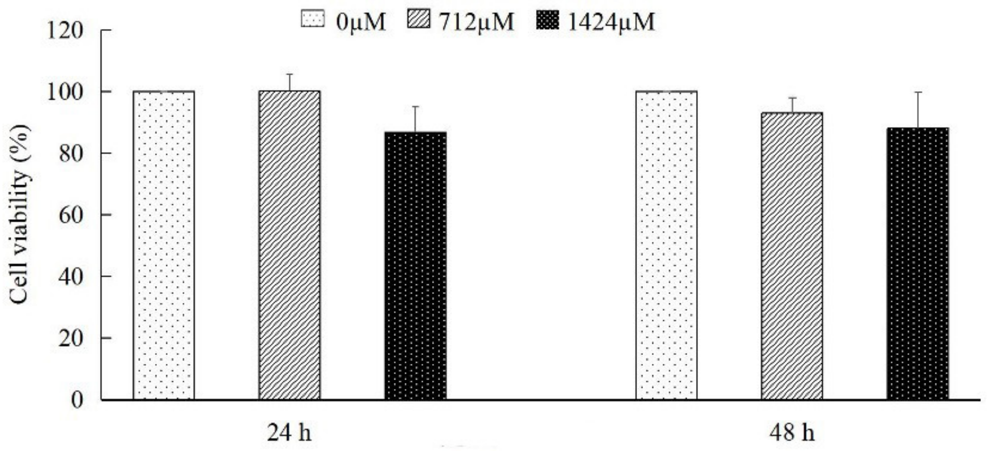

Figure S-3. Cytotoxicity and proliferation effects of DP9 for L929 cells.

S6. BxPC-3 cells invasion assay

Matrigel was thawed and diluted to 5 mg/mL in serum-free cold medium. 80 μL diluted Matrigel was added into the upper chamber. After incubation at 37 °C for 5 h, the gelled Matrigel was washed gently with warm serum-free medium. After 24 h

incubation, invasive cells were also photographed after fixing and staining.

In Figure S-5, the number of the invasion BxPC-3 cells were dramatically reduced with DP9 dose-dependent. Hence, DP9 significantly suppressed BxPC-3 cell invasion without affecting cell viability.

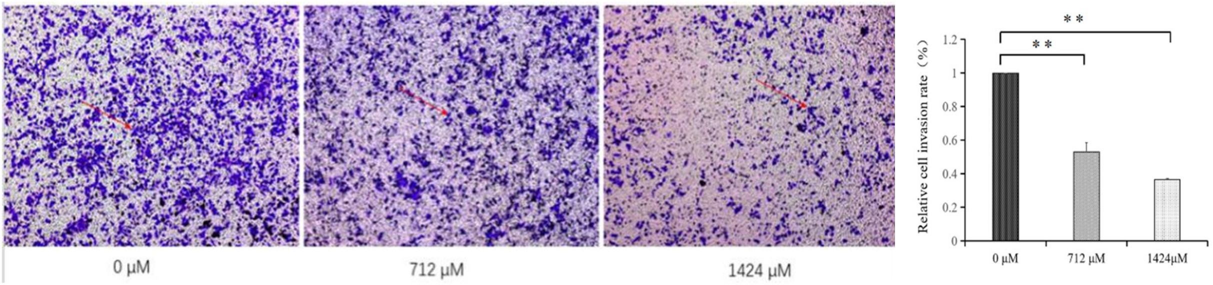

Figure S-4. Effect of DP9 on BxPC-3 cell invasion (Transwell assay with Matrigel coating). The number of invasive cells was significantly reduced in a dose-dependent manner with DP9 treatment, compared to the control group.

S7. BxPC-3 cells apoptosis assay

Logarithmic growth BxPC-3 cells were washed and suspended in a medium without serum. Then seeded at $2x10^6$ cells/well in a 12-well plate and treated with different doses of DP9 (0, 712, 1424 μM). After 24 h culture, the cells were harvested and washed with cold PBS and stained with PI/FITC-Annexin-V using an apoptosis detection kit according to the manufacturer's instructions.

To further analyze the cell apoptotic properties, BxPC-3 cells were treated with different concentrations of DP9 for 24 h. As shown in Figure S-4, blue fluorescent particles density in DP9-stimulated BxPC-3 cells was significantly higher than those without DP9. The results showed that DP9 induced BxPC-3 cells apoptosis in dose-dependence after 24 h treatment.

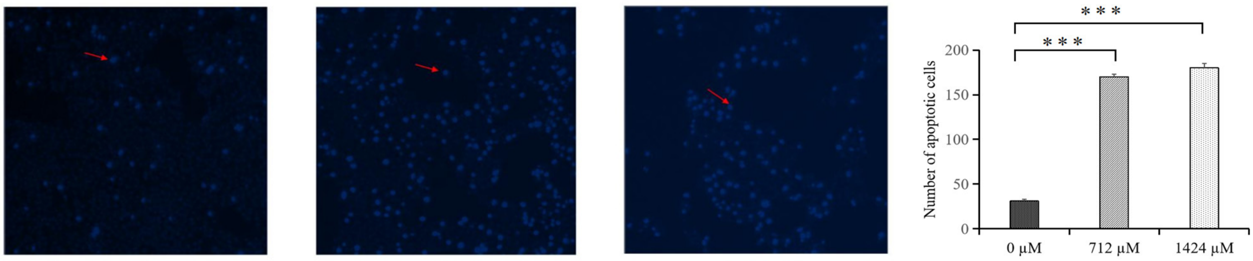

0 µM 712 µM 1424 µM

Figure S-5. Effect of DP9 on apoptosis of BxPC-3 cells and the number of apoptotic BxPC-3 cells under different concentrations of DP9, Fluorescent compounds used: PI (Propidium Iodide) is used to label necrotic cells and late apoptotic cells (red fluorescence), FITC-AnnexinV is used to label early apoptotic cells (green fluorescence), and double-positive cells are late apoptotic cells.

S8. Electrospray ionization mass spectrometry (ESI-MS) analysis

The instrument was equipped with a capillary electrophoresis interface following the configuration described by Costa et al. (2020), employing a triaxial flow arrangement where the capillary electrophoresis eluent mixed with a suitable sheath liquid at the probe tip before nebulization with nitrogen gas. Operating conditions included applied voltages of 120 kV for normal electro-osmotic flow and 220 kV for reversed electro-osmotic flow on the buffer reservoir, with an additional 3.5 kV ESI voltage applied at the separation column outlet. Characteristic peaks with relative intensities of 5-14% were observed at 46 m/z following base mass peaks, resulting from interactions between the sample and nebulization gas during the ionization process.
